# Synovial sarcoma of the ethmoid sinus with extension to the cavernous sinus: a case report and literature review

**DOI:** 10.1093/jscr/rjae579

**Published:** 2024-10-02

**Authors:** Haneen H Sharabati, Laila R Innab, Saja S Hussein, Ayman A Salman, Anis M Naser, Izzeddin A Bakri

**Affiliations:** Al Quds University Faculty of Medicine, Jerusalem, Palestine; Al Quds University Faculty of Medicine, Jerusalem, Palestine; Al Quds University Faculty of Medicine, Jerusalem, Palestine; Department of Neurosurgery, Al-Makassed Islamic Charitable Hospital, Jerusalem 00970, Palestine; Department of Neurology, Al-Makassed Islamic Charitable Hospital, Jerusalem 00970, Palestine; Department of Pathology, Al-Makassed Islamic Charitable Hospital, Jerusalem 00970, Palestine

**Keywords:** synovial sarcoma, ethmoid sinus, stroke, cavernous sinus, soft tissue

## Abstract

Synovial sarcomas are rare soft tissue tumors primarily affecting the extremities but can occasionally manifest in unusual locations such as the ethmoid sinus, posing diagnostic challenges. We present a case of a 38-year-old male with a 7-month history of recurrent throbbing headaches, left eye pain, and facial nerve palsy, evolving into multiple stroke episodes. Radiological studies showed extension to the cavernous sinus, raising an initial suspicion of vasculitis. Histological findings of an endoscopic biopsy, however, confirmed a monophasic synovial sarcoma. The patient was referred to a specialized center for further management. Unfortunately, he developed another stroke before receiving treatment. Management included chemotherapy and definitive radiation therapy targeting the ethmoid sinus. The patient is currently receiving ongoing palliative care for symptom management. This case underscores the importance of early diagnosis and a multidisciplinary approach in managing rare and aggressive tumors such as synovial sarcoma of the ethmoid sinus.

## Introduction

Synovial sarcomas are rare aggressive soft tissue tumors typically found in the extremities of young adults [1]. Their occurrence in the head and neck region is uncommon, and involvement of the paranasal sinuses is even rarer [2, 3]. The ethmoid sinus is the rarest site, with only five documented cases of female predominance reported in the English literature to date [4–7]. Here we present a case of synovial sarcoma affecting the ethmoid sinus in a young male, characterized by extension to the cavernous sinus and presenting with multiple strokes. Additionally, we provide a literature review to offer further insight into such rare cases.

## Case description

A 38-year-old Mediterranean man presented with a 7-month history of recurrent headaches, left eye pain, blurred vision, and left-sided facial weakness. The patient’s clinical course then evolved into a substantial 15 kg weight loss and recurrent strokes, characterized by right-sided weakness, dysarthria, and confusion.

Clinical examination revealed exophthalmos of the left eye and a mass occupying the left nasal cavity. Neurological examination revealed horizontal nystagmus when looking to the right, absent gag reflex, right-sided extremity weakness with hypertonia, and positive Babinski reflex.

**Figure 1:**
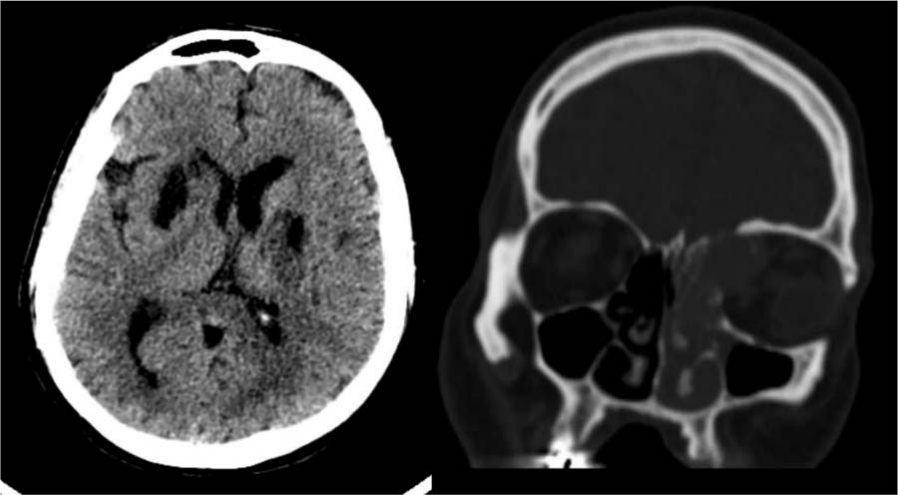
High-resolution CT scan with an axial cut (left side) showing a bilateral basal ganglia hypodense area suggestive of chronic ischemia and a nearby hypodense area at the left basal ganglia indicative of subacute ischemia (A). A coronal cut (right side) shows an iso-dense expansible soft tissue lesion with internal foci of calcifications centered in the anterior aspect of the left ethmoid sinus with protrusion to the left orbital conus pushing the medial rectus muscle laterally with complete obliteration of the left frontal sinus.

**Figure 2:**
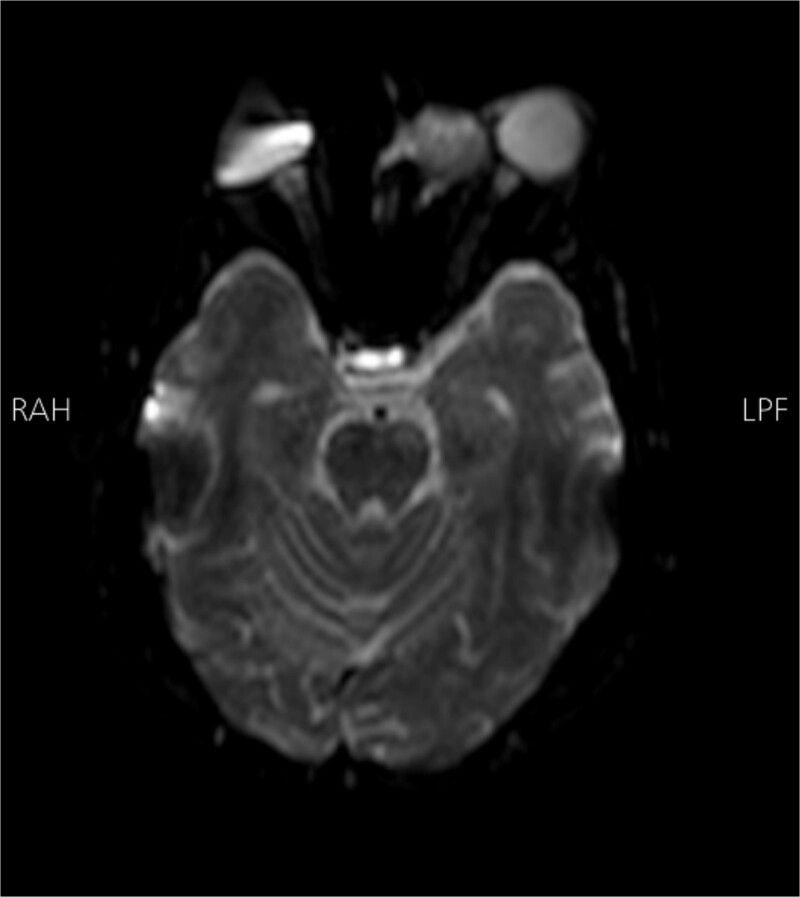
Brain MRI DWI sequence showing abnormal hyper-intense signal (bright) in the distribution of the left cavernous sinus. The left ethmoidal sinus synovial tumor is also noted.

Radiological findings from a high-resolution CT scan (Fig. 1) revealed bilateral basal ganglia hypodense areas suggestive of chronic ischemia and a more lateral hypodense area toward the left basal ganglia indicative of subacute ischemia. A coronal cut showed an iso-dense expansible soft tissue lesion with internal foci of calcifications centered in the anterior aspect of the left ethmoid sinus with protrusion to the left orbital conus, pushing the medial rectus muscle laterally with complete obliteration of the left frontal sinus. Brain MRI with FLAIR (Fig. 2) showed an abnormal hyperintense signal in the left cavernous sinus and a left ethmoidal sinus synovial tumor.

**Figure 3:**
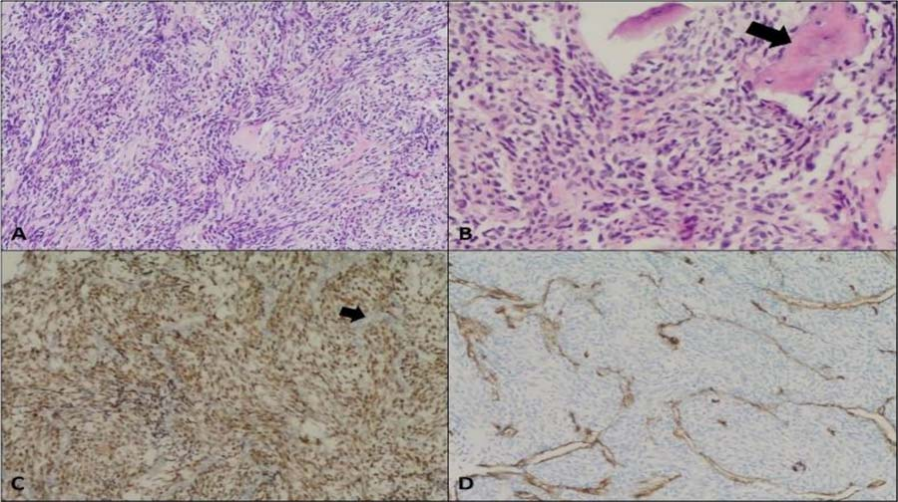
Synovial sarcoma, monophasic. (A) Moderately cellular neoplasm composed of monotonous oval to spindle-shaped cells (Hematoxylin and eosin stain, 10×); (B) Foci of bone trabeculae entrapment noted (arrow) (20×); (C) Positive TLE1 immunostaining (nuclear brown staining); negative internal control in endothelial cells (arrow) (10×); (D) Negative CD34 immunostaining in the neoplastic cells (highlight endothelial cells only). The tumor cells were negative for CKAE 1/3, EMA, CK7, CK19, S-100, Myogenin, SMA, Progesterone receptor, STAT6, and Beta-catenin.

Extensive vasculitis work-up yielded inconclusive results. Microscopic examination of an endoscopic biopsy sample (Fig. 3) depicted a monophasic synovial sarcoma composed of monotonous oval to spindle-shaped cells with minimal to moderate cytoplasm. Immunostaining for Transducin-like-Enhancer of split-1 (TLE1), B-cell lymphoma 2 (BCL2), and Cluster of Differentiation 99 (CD99) confirmed the diagnosis. Negative results for CD34, signal transducer and activator of transcription 6 (STAT6), S-100, epithelial membrane antigen (EMA), and cytokeratin AE (CKAE) effectively ruled out alternative pathologies. Our patient was referred to a higher-qualified center for further management. Unfortunately, he developed another stroke before receiving care, which worsened his right-sided weakness, and resulted in aphasia and urinary dysfunction. He then received four cycles of intra-arterial (IA) chemotherapy as neoadjuvant therapy and completed definitive radiation therapy (XRT) targeting the ethmoid sinus. A follow-up CT scan with IV contrast 1 month after treatment showed that the left ethmoid sinus remained unchanged compared with previous imaging, with left parietal encephalomalacia and faint contrast opacification of middle cerebral artery branches, likely related to subacute-chronic infarctions. The patient is currently receiving ongoing palliative care for symptom management.

## Discussion

Sarcomas are malignant tumors of connective tissue origin that favor the extremities, especially tendon sheaths and joint capsules, but can also arise elsewhere [8, 9]. Synovial sarcomas predominantly occur in young males in the second and third decades of life [1]. Involvement of the paranasal sinuses is rarely reported, with the ethmoid sinus being a rarer site for synovial sarcoma development. To date, five cases of ethmoidal sinus synovial sarcomas have been reported [4–7].

Clinically, most cases of ethmoidal sinus sarcomas present with nasal obstruction, pain, and epistaxis [10]. However, local invasiveness and spread to the cavernous sinus widens the presentation. Our patient presented with recurrent episodes of headache associated with blurry vision, dysarthria, and facial nerve palsy, suggesting recurrent attacks of stroke. Anatomically, this is explained by the venous drainage of the ethmoidal sinus through the superior ophthalmic vein into the cavernous sinus. From there, the involvement of the internal carotid artery may lead to the development of recurrent strokes.

A CT scan may indicate malignancy of an ethmoid sinus tumor, but its features often overlap with other paranasal sinus tumors. Histopathological examination is mandatory for confirmation. Sarcomas are classified based on the relative proportion of these two cellular components and the degree of differentiation [7, 11]. In this case, microscopic sections revealed monotonous oval to spindle-shaped cells with minimal to moderate cytoplasm. Differential diagnoses included squamous cell carcinoma, fibrosarcoma, leiomyosarcoma, malignant peripheral nerve sheath tumor, and monophasic synovial sarcoma. In our case, immunohistochemistry confirmed the diagnosis of monophasic synovial sarcoma, with the tumor cells being non-reactive for S-100, Smooth Muscle Actin (SMA), and neurofilament, but positive for TLE1, BCL2, and CD99, which are indicative of this type of sarcoma [12].

Treatment involves wide local excision of normal tissue, followed by chemo-radiation [12, 13]. However, achieving negative margins can be challenging with paranasal sinus surgery, especially when performed endoscopically. Therefore, in advanced cases, an open surgical approach is preferred. Complete resection can be confirmed by taking a frozen section after complete resection of the tumor [14].

Postoperative radiation therapy can improve local control and increase overall survival rates in sarcomas [15]. A retrospective analysis by Andra et al. found that among 26 patients with head and neck sarcomas who received postoperative radiation therapy, 96% of patients had high-grade lesions. Among those who underwent surgery, 38% had tumor-free margins, 23% had microscopically positive margins, and 19% had gross residual disease. All cases received 66 Gy radiotherapy, with 50% receiving sequential chemotherapy [15]. Five-year local control was 86%, while overall survival was 82%. However, the prognosis remains poor even with extensive management [13]. Table 1 summarizes previously reported cases of ethmoid synovial sarcoma, highlighting patient demographics, presentations, and outcomes.

**Table 1:** Summary of cases of synovial sarcoma of the ethmoid sinus and their outcomes

**Author**	**Age/sex**	**Presentation**	**Treatment**	**Outcome**
Kartha [7]	24/F	Pain and proptosis	Open resection followed by chemotherapy and radiotherapy after recurrence	Recurrence after 1 month, with metastasis to the lung and brain. Died at 9 months.
Wong [4]	80/F	Persistent right-sided epistaxis	Endoscopic excision followed with radiotherapy after recurrence	Recurrence after 3 months with metastasis to the brain and lymph nodes. Died at 9 months.
Jain [5]	36/F	Left nasal obstruction and epistaxis.	Endoscopic excision followed with postoperative radiotherapy.	No evidence of disease at 6 months.
Sapna1 [6]	43/F	Left nasal obstruction with swelling in the medial canthal region.	Endoscopic resection followed with postoperative chemotherapy (Ifosfamide) and radiotherapy	Recurrence after 6 months.
Sapna2 [6]	82/F	Right nasal obstruction, epistaxis, and external nasal deformity	Chemotherapy (Ifosfamide).	Died after 6 weeks.
Present case	38/M	Headache and recurrent strokes	Four cycles of IA neo-adjuvant chemotherapy, with definitive radiation therapy targeting the ethmoid sinus.	Follow up after 1 year showed: right-sided weakness, aphasia, wheelchair-bound. Currently receiving palliative care.

## Conclusion

Synovial sarcoma of the ethmoid sinus is extremely rare and presents significant clinical challenges due to its potential for local invasion and systemic effects. This case highlights the rarity and complexity of synovial sarcoma in the ethmoid sinus, emphasizing the need for early diagnosis. Effective management requires a combination of surgery, chemotherapy, and radiotherapy. Continuous follow-up and palliative care are essential to address the ongoing needs of patients. Further research is required to improve understanding and outcomes for these patients.
